# A New Leaf

**Published:** 1873-01

**Authors:** 


### HALL'S

### JOURNAL OF HEALTH

Vol. XX.—JANUARY, 1873.—No. I.

### A NEW LEAF

For this first day of a new year may well be turned by all who read these lines, to the end of preserving health, renewing life, and adding largely to the sum total of human elevation and human happiness.

The wretched dyspeptic may turn a new leaf to infinite advantage, by eating half as much as he now does, at three regular times a day, and spending four hours out of every twenty-four in agreeable and profitable out-door activities.

The drunkard's new leaf should be the resolution to never touch another drop of liquor, and cure himself ot the love of it by taking the following preparation twice a day as an effectual means of preventing that most distressing physical and mental prostration inseparable from the sudden abandonment of an accustomed stimulant; it has cured thousands of drunkards, and will cure thousands more if they want to be cured :—Sulphate of iron (that is, copperas), five grains; magnesia, ten grains; spirit of nutmeg, one dram; and peppermint water, eleven drams or teaspoonfuls. This should be taken night and morning.

The tobacco-user should turn his new leaf, and no more say,

> "Three trades I carries on without any flaws:
> I smokes, I snuffs, I chaws."

Let the young clerk turn over his new leaf by devoting himself so completely to his employer's interests, as to make him feel that he cannot do without him; hence either raises his salary, or takes him in as a partner.

A new leaf for a young lady is the discouragement of all advances for her hand on the part ot any young man who speaks disrespectfully of religion, ot the Bible, or of womankind.

The mechanic's new leaf is to do his work as thoroughly and as well for his employer as he would for himself; such a man will always find plenty of work to do, at good prices.

Here is a new leaf for a merchant: Ask what you please for your goods, but never represent them to be of a better quality than they really are.

The doctor's new leaf should be, never to give medicine to a patient which he would not take himself under the same circumstances.

The lawyer's leaf is to counsel friendly arbitration instead of belligerent law, but charge the same fee.

The clergyman's best leaf is to preach every sermon as if it were the last opportunity he will ever have again of performing the great work of his life.

A young man may turn an important new leaf by the resolution to never take a grain or a drop of anything which can intoxicate.

The mistress may make her new leaf add to her comfort in the long run, by remembering that her servants are brimful of human nature and the old Adam; and that one of the best ways of managing them is to treat them courteously, kindly and considerately, but never allow them to shirk their work or be impudent.

The servant may turn a new leaf by performing every duty promptly, thoroughly and well.

A new and good leaf for the mother to turn would be a resolution never to reprove a child in the presence of a third person, or on the spur of the moment, but wait until alone together, and until next day.

The father may turn his leaf by making his children his companions, and getting into the way of consulting them about matters and things; this sets them to thinking; gives them encouragement, and helps them to self-confidence, and self-reliance, and manliness.

A son's new leaf should be, never to cross his father's wishes.

The good daughter will resolve to help her mother all she can, in every possible way.

The old man may turn a leaf for the better by resolving to grow more kindly every day.

The old woman's best leaf is in kindly and impressive ways to teach her children and grandchildren that, to be patient and to make allowance for the failings of others, will add much to their own happiness and those with whom they may be brought in contact.

The husband's new leaf should be to remember that the wife of his bosom and the mother of his children is entitled to the first place in his own affections, to the first place in the obedience of the child; to the first place in the respect of every servant, and to the highest seat of honor in all the circumstances of life.

The wife may present her new leaf to her husband by sewing on his buttons and darning his stockings promptly and well, to the end that his clothing shall be always ready for instant use.

The creditor's new leaf should be to be considerate; help rather than push; encourage rather than insult.

The debtor owes a new leaf to the man who has trusted him, to resolve that a dollar shall never lie idle in his house an hour, if he knows his creditor really needs that dollar.

And let every subscriber to a paper or magazine remember that prepayment is simple justice; and that every day a subscription is deferred adds to the wrong done the publisher; and if you do a deliberate wrong to-day, and repeat it to-morrow, in a small thing, you are in danger of stealing if it were certain you would not be found out; the very smallness of the debt increases the enormity of the injustice of withholding what is due, because you can so easily pay it, while that very sum—a single dollar though it be—may be just the amount needed to complete the sum necessary to take up a note in bank, which, if not done, credit is ruined, and a man's business is broken up. Such things have occurred before, and will assuredly occur again, but "It will not be on my account," says the generous reader. We are not talking for ourselves, for no man owes the JOURNAL a dollar, nor will have the chance of doing it.

And now let the one magnificent leaf be turned over on the last hour of the last day of old eighteen hundred and seventy-two—turned over by all creation, as to mundane affairs.

### PAY AS YOU GO.

If you cannot pay on the spot for what you want, have the independence to do without it, even if you have to lie in bed a whole day for want of fire, or have to live on bread and water for a week.

Try it, reader, for one year, never to incur the debt of even a dollar, and pay what you owe now, as fast as you can, the smallest debts first; and when seventy-three closes you will be a healthier, happier, and a better man, so in love with "the principle of the thing" that you will never go in debt again as long as you live

